# Antimicrobial resistance patterns of Uropathogens isolated from adult women with acute uncomplicated cystitis

**DOI:** 10.1186/s12866-019-1612-6

**Published:** 2019-10-30

**Authors:** Jamaan Al-Zahrani, Khaled Al Dossari, Ahmed H. Gabr, Abul-fotouh Ahmed, Saad Abdulrahman Al Shahrani, Sameer Al-Ghamdi

**Affiliations:** 1 Department of Family Medicine, College of Medicine, Prince Sattam bin Abdulaziz University, Al Kharj 11942, Saudi Arabia; 2 Department of Urology, Minia University, Minia, Egypt; 3 Department of Urology, Al-Azhar University, Cairo, Egypt; 4 Department of Urology, College of Medicine, Prince Sattam bin Abdulaziz University, Al Kharj 11942, Saudi Arabia

**Keywords:** Antibiotic, Cystitis, Resistance, Uropathogen

## Abstract

**Background:**

To evaluate the antibiotic resistance patterns of uropathogens isolated from adult women with acute community-acquired (CA) uncomplicated cystitis.

**Results:**

Over a one-year period (May 2015–April 2016), the results of susceptibility testing of outpatient midstream urine samples from 5 different laboratories were prospectively evaluated. The study included only adult women with uncomplicated cystitis. The susceptibility testing in all laboratories was performed using the disk diffusion method with the VITEK-2 Compact system. The isolated uropathogens and their resistance to the tested antibiotics were evaluated. Out of 317 adult women with CA uncomplicated cystitis, 179 had a positive culture. The most commonly isolated organism was *Escherichia coli* (*E. coli*) (70.4%), followed by *Klebsiella* (21.2%). The overall resistance rate was highest for ampicillin (85.6%), followed by cefalotin (56.3%), trimethoprim/sulfamethoxazole (54.7%), pipracillin (51.9%), nitrofurantoin (48.8%) and aztreonam (47.4%). Isolated *E. coli* strains were commonly resistant to ampicillin (80.5%), trimethoprim/sulfamethoxazole (72.2%) and aztreonam (71.4%), followed by cefalotin (55.9%). The overall ciprofloxacin resistance rate was 17.9%, and the resistent was found only with *E. coli* (25.4%).

**Conclusions:**

Our results may aid in the selection of proper empiric antibiotic therapy for adult women with acute CA uncomplicated cystitis.

## Background

Urinary tract infection (UTI) is one of the most common bacterial infections seen in women. More than 50% of women develop at least one episode of UTI at some point in their lives, and many will have recurrences [1]. Although most UTIs are less severe, such infections can cause patients significant distress and are associated with high healthcare costs and social burden [2]. In adult women, acute community-acquired (CA) uncomplicated cystitis accounts for the majority of UTI and community use of antibiotics. Moreover, *Escherichia coli* (*E. coli*) is the most commonly isolated pathogen in uncomplicated cystitis, accounting for 75–90% of isolates in the outpatient setting [3].

The diagnosis of acute CA uncomplicated cystitis is usually based mainly on clinical manifestations [4, 5]. Clinicians generally prescribe empiric antibiotic therapy for uncomplicated cystitis and recommend microbial identification and susceptibility testing only in cases of recurrent UTI (rUTI), resistant infections or failure of empiric therapy [5, 6]. The rationale for empiric antibiotic therapy is based on the predictable and narrow spectrum of the causative organisms and their susceptibility patterns. However, the misuse of antibiotics is the main origin of acquired microbial resistance to commonly prescribed antibiotics. Because of the rise in microbial resistance over time and potential differences in antibiotic susceptibility in different countries, routine assessment of antibiotic susceptibility patterns in different geographical regions is necessary to facilitate the selection of empiric therapy. The current study was conducted to determine the local antimicrobial susceptibility patterns of common uropathogens isolated from urine cultures of women with acute CA uncomplicated cystitis. The aim of the study was to identify alternative empiric antibiotic therapies that may be effective against common uropathogens in Riyadh, Saudi Arabia.

## Results

Over the one-year period, a total of 2073 culture reports from outpatient urine samples were available. From those, only 317 urine samples were from adult women with CA uncomplicated cystitis. Of the 317 urine cultures, 179 (56.5%) were positive with available sensitivity reports and were included in the data analysis.

The age of the participants ranged from 18 to 70 years (median: 28 years). The isolated uropathogen were *E. coli* (70.4%), *Klebsiella* (21.2%), *Enterobacter* (3.4%), *Proteus* (2.8%), *Pseudomonas* (1.7%) and *Cedecea* (0.6%). Stratified by age, the most commonly isolated uropathogen in women aged ≤50 years and those > 50 years was *E. coli* (70.8% vs. 66.7%) and *Klebsiella* (19.9% vs. 33.3%). All other uropathogens, except for *E. coli* and *Klebsiella*, were isolated only from women ≤50 years of age. No significant difference was found between both age groups for the distribution of isolated uropathogens (*P* = 0.685).

All isolated uropathogens were resistant to at least two of the tested antibiotics. Overall, the rate of antibiotic resistance was highest for ampicillin (85.6%), cefalotin (56.3%), trimethoprim/sulfamethoxazole (54.7%), pipracillin (51.9%), nitrofurantoin (48.8%) and aztreonam (47.4%). The overall resistance rates to ciprofloxacin and levofloxacin were 17.9 and 10.5%, respectively. Among *E. coli* isolates, 80.5% were resistant to ampicillin, 72.2% to trimethoprim/sulfamethoxazole, 71.4% to aztreonam and 55.9% to cefalotin. The antibiotic susceptibility pattern for *E. coli* also demonstrated high sensitivity to nitrofurantoin, ciprofloxacin and levofloxacin. The antibiotic resistance patterns of isolated uropathogens to the tested antibiotics are shown in Table 1.

**Table 1:** Antibiotic resistance of the isolated organisms to the tested antibiotics

Tested antibiotics	Isolated organisms						
	Overall	*E.coli*	*Klebsiella*	*Enterobacter*	*Proteus*	*Pseudomonas*	*Cedecea*
Antibiotics tested against lactose and non-lactose fermenting organisms							
	(*n* = 179)	(*n* = 126)	(*n* = 38)	(*n* = 6)	(*n* = 5)	(n = 3)	(n = 1)
Amikacin	18 (10.1)	18 (14.3)	0 (0.0)	0 (0.0)	0 (0.0)	0 (0.0)	0 (0.0)
Cefepime	36 (20.1)	29 (23.0)	6 (15.8)	0 (0.0)	0 (0.0)	0 (0.0)	1 (100)
Ceftazidime	36 (20.1)	29 (23.0)	6 (15.8)	0 (0.0)	0 (0.0)	0 (0.0)	1 (100)
Ciprofoxacin	32 (17.9)	32 (25.4)	0 (0.0)	0 (0.0)	0 (0.0)	0 (0.0)	0 (0.0)
Gentamicin	32 (17.9)	26 (20.6)	6 (15.8)	0 (0.0)	0 (0.0)	0 (0.0)	0 (0.0)
Imipenem	6 (3.4)	0 (0.0)	6 (15.8)	0 (0.0)	0 (0.0)	0 (0.0)	0 (0.0)
Meropenem	3 (1.5)	0 (0.0)	0 (0.0)	0 (0.0)	0 (0.0)	0 (0.0)	0 (0.0)
Tigecycline	18 (10.1)	0 (0.0)	6 (15.8)	6 (100)	5 (100)	0 (0.0)	1 (100)
Pipracillin/Tazobactam	25 (14.0)	18 (14.3)	6 (15.8)	0 (0.0)	0 (0.0)	0 (0.0)	1 (100)
Trimethoprim/Sulfamethoxazol	98 (54.7)	71 (72.2)	6 (15.8)	0 (0.0)	0 (0.0)	0 (0.0)	1 (100)
Antibiotics tested against lactose fermenting organisms							
	(*n* = 160)	(*n* = 118)	(*n* = 36)	(n = 6)	(*n* = 0)	(n = 0)	(n = 0)
Amoxacillin/Clavulanic acid	66 (41.3)	54 (45.8)	6 (16.7)	6 (100)	–	–	–
Ampicillin	137 (85.6)	95 (80.5)	36 (100)	6 (100)	–	–	–
Cefalotin	90 (56.3)	66 (55.9)	18 (50.0)	6 (100)	–	–	–
Cefoxitin	48 (30.0)	36 (30.5)	6 (16.7)	6 (100)	–	–	–
Ceftriaxone	48 (30.0)	36 (30.5)	6 (16.7)	6 (100)	–	–	–
Nitrofurantoin	78 (48.8)	36 (30.5)	36 (100)	6 (100)	–	–	–
Pipracillin	83 (51.9)	59 (50.0)	24 (66.7)	0 (0.0)	–	–	–
Antibiotics tested against non-lactose fermenting organisms							
	(*n* = 19)	(*n* = 8)	(*n* = 2)	(n = 0)	(n = 5)	(n = 3)	(n = 1)
Ampicillin/Sulbactam	2 (10.5)	1 (12.5)	0 (0.0)	–	0 (0.0)	0 (0.0)	1 (100)
Aztreonam	9 (47.4)	5 (62.5)	0 (0.0)	–	0 (0.0)	3 (100)	1 (100)
Colistin	8 (42.1)	0 (0.0)	0 (0.0)	–	5 (100)	3 (100)	0 (0.0)
Ertapenem	3 (15.8)	2 (25.0)	0 (0.0)	–	0 (0.0)	1 (0.0)	1 (100)
Levofloxacin	2 (10.5)	2 (25.0)	0 (0.0)	–	0 (0.0)	0 (0.0)	0 (0.0)
Minocycline	8 (42.1)	2 (25.0)	0 (0.0)	–	5 (100)	0 (0.0)	1 (100)
Ticarcillin/Clavulanic acid	8 (42.1)	2 (25.0)	0 (0.0)	–	5 (100)	0 (0.0)	1 (100)

Data presented as number (percentage)

## Discussion

Inappropriate use of antibiotics, excessively frequent prescription of broad-spectrum antibiotics and inadequate use of antibiotics by patients are the leading causes of bacterial gene mutations and subsequent resistance to antibiotics [7]. The study by Baddour and colleagues [8] revealed that the trend in the susceptibility pattern of methicillin-resistant *Staphylococcus aureus* towards antibiotics had increased to 33.8% with sulfamethoxazole/trimethoprim and 39.6% with gentamicin. Moreover, local studies demonstrated a high prevalence of multiple drug-resistant organisms and the development of extended-spectrum beta-lactamase pathogens [9].

Resistance to antimicrobial agents is considered a significant global challenge. This might be attributed to the fact that most CA-UTIs are treated without bacteriological testing. Understanding the antibiotic susceptibility patterns of causative organisms will result in a better selection of antimicrobials given as empiric therapy for UTIs.

There is a significant geographic discrepancy in in vitro susceptibility for *E. coli*. In four different studies, resistance rates were higher in Spain and Portugal than other European countries, and medical centers in the United States showed higher resistance rates than medical centers in Canada [10, 11]. The present study reports antibiotic resistance patterns of women with CA uncomplicated cystitis in our region. Most studies have identified *E. coli* as a main causative pathogen of UTI [12, 13]. Our data confirmed that *E. coli* was indeed the most common causative organism of UTI (70.4%) in our region.

The presence of Klebsiella as a causative pathogen of CA-UTI was variable among different studies. In a study from India [14] the incidence was reported to be 22%, while in another study from Kuwait [15] the incidence was 10.8%. In the present study, *Klebsiella* was the second most commonly isolated uropathogen accounting for 21.2% of cases in the studied population.

In our study, the resistance rate to ampicillin among *E. coli* isolates was 80.5%. This is considered very high compared with previous studies that showed an ampicillin resistance rate of 30% in Canada [16] and 49% in the United Kingdom [17]. In a surveillance program, the ECO.SENSE project [18], the resistance of *E. coli* to ampicillin was refined according to its relation with resistance to other antimicrobials. Kahlmeter et al. [5] reported a resistance rate of 80% to ampicillin among ciprofloxacin-resistant *E. coli* strains, which is comparable to our results. For trimethoprim/sulfamethoxazole resistance, our results also indicated a much higher resistance than previous studies (11% in Canada and 16–18% in the United States) [16]. This difference might be attributed to the ease of obtaining these antibiotics from the drug market in our country compared with tight restrictions enforced in other countries. Our finding of increased resistance to ampicillin and trimethoprim/sulfamethoxazole suggests that these agents may no longer be suitable for empiric therapy against CA-UTI. Our data showed that individual antibiotic activity was lower compared with antibiotic combinations with β-lactamase inhibitors, such as sulbactam and tazobactam, which may suggest that these bacteria may contain β-lactamase enzymes.

Fluoroquinolones have been suggested as reasonable empiric treatment for CA-UTI [13]. With a resistance rate of 17.9% for ciprofloxacin and 10.5% for levofloxacin in our study, this could be plausible. However, the increasing use of this antimicrobial group raises concerns regarding the possibility of increasing resistance patterns in the near future. Our concerns are further validated by the recent international clinical practice guidelines, which recommended fluoroquinolones as an alternative agent for the treatment of uncomplicated UTIs, rather than the previously recommended first-line therapy [19]. Additionally, this is in agreement with the recent announcement of the US Food and Drug Administration. The package insert of fluoroquinolones will be updated to include a warning stating that this antimicrobial agent should not be used for routine respiratory tract infections or uncomplicated UTIs unless there is no suitable alternative agent [20].

Isolated *E. coli* strains were found to be the most susceptible to imipenem, meropenem, tigecycline and colistin. The apparent lack of resistance to these antibiotics may be explained by its limited availability in our region and its high cost.

There are some limitations associated with this study. First, the study population was limited to one locality. A larger cohort of patients from different geographical regions with different health conditions with study of the mechanisms through which bacteria develop resistance may provide more informative data. Second, the results of our study were based on culture reports from five different laboratories with different laboratory procedures, which might have led to variations in resistance rates. Finally, possible side effects associated with each antimicrobial agent were not reported. However, such side effects were not anticipated to affect the rates of antimicrobial resistance.

## Conclusions

*E. coli* remains the most commonly isolated Gram-negative causative organism of UTI, followed by *Klebsiella*. Isolated uropathogens demonstrated high sensitivity to imipenem, meropenem, Ampicillin/Sulbactam and levofloxacin. As an oral and cost-effective therapy, we recommend that levofloxacin be used as alternative antimicrobial treatment for CA uncomplicated cystitis. Further studies with a larger number of patients in different regions should be performed to recommend national guidelines for the treatment of CA-UTI.

## Methods

Data were collected from 5 clinical microbiology laboratories and in the province of Riyadh, Saudi Arabia, during the one-year period between May 2015 and April 2016. All patients were referred to the laboratories either by Urologists or primary health care physicians at two University hospitals and three primary health care centers. Patients’ medical files were reviewed to limit the analysis to CA uncomplicated cystitis. Adult women (at least 18 years of age) who presented at outpatient clinics with symptoms of acute cystitis (dysuria, urgency, frequency, hematuria, or suprapubic pain) and agreed to participate in the study were prospectively recruited. Women with a history of rUTI (defined as three or more UTI episodes in the past year or two or more episodes in the past 6 months), recent antibiotic treatment within the past two weeks, immunocompromised state, pregnancy, urinary catheter, associated urinary tract pathology or anatomical abnormalities and those with history of hospitalization within the past month were excluded. Ethics approval for the study project was obtained from the Deanship of Scientific Research at Prince Sattam Bin Abdulaziz University. All study procedures were performed in accordance with the principles laid down in the Helsinki Declaration (1975) and its later amendments.

A midstream urine sample was collected by each participant in a sterile container and sent to the main laboratory within 20 min for culture and sensitivity analysis and processed within 2 h. Cultures that showed atypical organisms and mixed organisms suggestive of contamination were excluded from the analysis. A urine culture was considered to be positive for infection if the colony count for a single organism was more than 10^5^ colony-forming units/ml.

All isolates were identified and tested for susceptibility by the Vitek 2 system (bioMérieux; Inc., USA) using the Gram-negative strain cards AST-N291 and AST-P580. The isolates were tested for susceptibility for the following antimicrobial agents: amikacin, cefepime, ceftazidime, ciprofloxacin, gentamycin, imipenem, meropenem, tigecycline, pipracillin/tazobactam, trimethoprim/sulfamethoxazole, amoxicillin/clavulanate, ampicillin, cefalotin, cefoxitin, ceftriaxone, nitrofurantoin, pipracillin, ampicillin/sulbactam, aztreonam, colistin, ertapenem, levofloxacin, minocycline, ticarcillin/clavulanic acid. The cards were inoculated and incubated in the system according to the manufacturer’s instructions. All results were interpreted using the Advanced Expert System (software version VT2-R04.03). Using the Vitek 2 system AST-N0291 card, the isolates were initially considered positive for extended spectrum beta-lactemase (ESBL) if the minimum inhibitory concentration of ceftazidime and cefotaxime for these organisms was ≥2 mg/L.

The proportions of overall resistance and individual resistance of isolated uropathogens were calculated for each of the tested antibiotics. Differences in the distribution of isolates by age groups were determined using the chi-square test or Fisher’s exact test. Differences were considered significant at *P* values of < 0.05. Statistical analyses were performed using SPSS version 25 (SPSS Inc., Chicago, IL).

## Data Availability

Data are available upon request from the authors.

## Abbreviations

CA: Community-acquired
*E. coli*: *Escherichia coli*
rUTI: Recurrent urinary tract infection
UTI: Urinary tract infection
